# 866. Evaluation of Alternative Predictors of Mortality in Burn Patients Controlled for Age and TBSA

**DOI:** 10.1093/jbcr/irag033.336

**Published:** 2026-04-06

**Authors:** Tiffany Shi, Trent James, Adam Hutchinson, Andrew Villegas, Julia C Slater

**Affiliations:** University of Cincinnati College of Medicine, Cincinnati, OH; University of Cincinnati College of Medicine, Cincinnati, OH; University of Cincinnati College of Medicine, Cincinnati, OH; University of Cincinnati College of Medicine, Cincinnati, OH; The University of Kansas Health System Burnett Burn Center, Kansas City, KS

## Abstract

**Introduction:**

Predictors of mortality in burn patients, including age, total body surface area (TBSA) of burn, and inhalation injury, are well-established components of scoring systems like the revised Baux (rBaux) score. However, outcomes still vary despite similar rBaux scores. This study explores hematologic and clinical parameters as predictors of mortality, focusing on patients controlled for age, TBSA, and rBaux scores to limit influence of these factors.

**Methods:**

Retrospective analysis included 35 patients ages 30-70 years with TBSA 30-70% surviving >4 days post-burn injury with ≥4 days in the intensive care unit. Associations between mortality and coagulation markers (PT, INR), hematologic parameters (white blood cell, platelets, and absolute neutrophils, lymphocytes, and monocytes), and differential blood count ratios (NLR, NMR, LMR) were assessed using labs nearest to time of admission. R software (v.4.2.0) was used for statistical analyses.

**Results:**

Of the included 35 patients, 24 (68.6%) survived and 11 (31.4%) died. Age, TBSA, and rBaux scores were appropriately controlled with no significant differences between groups. Univariate logistic regression showed that absolute monocyte count trended toward higher odds of mortality (OR 3.18, *p*=.062) and point-biserial correlation showed a positive association between mortality and monocyte counts (r = 0.409, *p*=.038). However, in a multivariable model, monocytes were not independently, but trended toward, significant (OR 3.50, *p*=.074). Correlation analyses revealed positive association between mortality and absolute monocyte counts (r = 0.409, *p*=.04). Length of hospital stay was negatively correlated with absolute monocyte counts (r = −0.406, *p*=.040). Of all patients, wound infection occurred in 7 patients (20%) and was associated with longer LOS (*p*=.013) but not mortality (*p*=1.00). Intriguingly, lower monocyte counts trended toward slightly higher odds of wound infection (OR 0.14, *p*=.068).

**Conclusions:**

When age and TBSA were controlled, differential blood count data, in particular absolute monocyte counts, emerged as potential predictors of mortality in burn patients. These findings suggest non-traditional parameters could complement established predictors to differentiate burn outcomes.

**Applicability of Research to Practice:**

Admission monocyte counts may help identify higher-risk burn patients, supporting closer monitoring, proactive infection prevention, and timely family counseling.

**Funding for the study:**

N/A.

